# Characterization of an Antiviral Component in Human Seminal Plasma

**DOI:** 10.3389/fimmu.2021.580454

**Published:** 2021-02-19

**Authors:** Ran Chen, Wenjing Zhang, Maolei Gong, Fei Wang, Han Wu, Weihua Liu, Yunxiao Gao, Baoxing Liu, Song Chen, Wei Lu, Xiaoqin Yu, Aijie Liu, Ruiqin Han, Yongmei Chen, Daishu Han

**Affiliations:** ^1^ Institute of Basic Medical Sciences, School of Basic Medicine, Peking Union Medical College, Chinese Academy of Medical Sciences, Beijing, China; ^2^ Department of Immunology, Shenzhen University School of Medicine, Shenzhen, China; ^3^ Department of Andrology, China-Japan Friendship Hospital, Beijing, China; ^4^ Science and Technology Innovation Center, Guangzhou University of Chinese Medicine, Guangzhou, China

**Keywords:** seminal plasma, prostate fluid, antiviral factor, mumps virus, sexual transmission

## Abstract

Numerous types of viruses have been found in human semen, which raises concerns about the sexual transmission of these viruses. The overall effect of semen on viral infection and transmission have yet to be fully investigated. In the present study, we aimed at the effect of seminal plasma (SP) on viral infection by focusing on the mumps viral (MuV) infection of HeLa cells. MuV efficiently infected HeLa cells *in vitro*. MuV infection was strongly inhibited by the pre-treatment of viruses with SP. SP inhibited MuV infection through the impairment of the virus’s attachment to cells. The antiviral activity of SP was resistant to the treatment of SP with boiling water, Proteinase K, RNase A, and DNase I, suggesting that the antiviral factor would not be proteins and nucleic acids. PNGase or PLA2 treatments did not abrogate the antiviral effect of SP against MuV. Further, we showed that the prostatic fluid (PF) showed similar inhibition as SP, whereas the epididymal fluid and seminal vesicle extract did not inhibit MuV infection. Both SP and PF also inhibited MuV infection of other cell types, including another human cervical carcinoma cell line C33a, mouse primary epididymal epithelial cells, and Sertoli cell line 15P1. Moreover, this inhibitory effect was not specific to MuV, as the herpes simplex virus 1, dengue virus 2, and adenovirus 5 infections were also inhibited by SP and PF. Our findings suggest that SP contains a prostate-derived pan-antiviral factor that may limit the sexual transmission of various viruses.

## Introduction

Many types of viruses can be detected in human semen, which poses a risk for the sexual spread of pathogens (1, 2). While 17 of 32 viruses present in semen are sexually transmissible (3), the efficiency of sexual transmission is variable. The efficiency of viral transmission is generally associated with seminal viral load and tropism for the ano-genital tract, and the mucosal barrier plays an important role in restricting sexual transmission of viruses (4). Growing evidences indicate that human semen may impact viral infection (5), which has yet to be intensively investigated.

Studies on the role of SP in viral infection have thus far shown conflicting results. Early studies showed that human seminal plasma (SP) significantly inhibited HIV-1 infection (6), and that multiple cationic polypeptides in SP played an anti-HIV-1 role (7). Seminal exosomes inhibit HIV-1 infection by blocking the interaction between HIV-1 and target cells (8–11). Recent studies showed that SP also inhibited ZIKV and cytomegalovirus (HCMV) infections (12, 13). In contrast, human semen-derived amyloid fibrils enhance HIV-1 infection, herpes simplex virus (HSV), HCMV, and EBOV infections (14–17). Moreover, SP may differentially impact HIV-1infection in a cell-specific manner (18), and the inflammatory status of the male genital tract can alter SP inhibition of HIV-1infection (19). These previous observations suggest that different mechanisms underlie the effect of SP on viral infection and are worthy of clarification.

MuV is the causative pathogen of mumps, a contagious disease worldwide (20). MuV also has a high tropism for the testis and induces orchitis, an etiologic factor of male infertility (21). MuV can be detected in mumps orchitis (22). MuV can efficiently replicate in most types of testicular cells and thereby perturb testicular functions (23–27). Sialic acid and Axl/Mer receptor tyrosine kinases play roles in MuV infection of testicular cells (28). In fact, MuV can infect various cell types *via* its receptor α2,3-sialic acid (29, 30). While MuV infects various cells of the male genital tract, it is not sexually transmitted. In the present study, we hypothesized that SP inhibits viral infection, and we attempted to elucidate the antiviral effect of SP on different cell types against various viruses. We found that SP potently inhibited MuV infection of different cell types of the genital tract and the infection of HeLa cells by various virus types, including MuV, herpes simplex virus 1 (HSV-1), adenovirus 5 (AV-5), and dengue virus 2 (DeV-2).

## Materials and Methods

### Cell Culture

Vero E6 (CRL-1586), HeLa (CCL-2), and 15P1 (CRL-2618) cell lines were purchased from ATCC (Manassas, VA, USA). C33A (HTB-31) cell line was obtained from the cell center of the Peking Union Medical College (PUMC, Beijing, China). The primary epididymal epithelial cells (EEC) were isolated from 3-week-old C57BL/6J mice based on previous described procedures (31). The cells were cultured in Dulbecco's modified Eagle’s medium (DMEM) (Thermo Fisher Scientific, Waltham, MA, USA) and supplemented with 5% fetal calf serum (FCS) (Thermo Fisher Scientific), 1.2 mg/ml sodium bicarbonate, 100 U/ml penicillin, and 100 mg/ml streptomycin, under a humidified atmosphere of 5% CO_2_ in compressed air at 37°C.

### Sample Collection

All of the human samples were obtained from the Andrology Department of the China-Japan Friendship Hospital. We collected 20 semen samples from healthy individuals between 30 and 40 years of age. After liquefaction at 37°C for 1 h, SP was obtained by centrifugation of semen at 12,000 × g for 10 min and collection of the cell-free supernatant. The SP was then stored at −80°C as individual or pooled samples. Ten prostatic fluids (PF) were obtained from healthy individuals 30 to 40 years of age. The prostate was massaged by urologist using a finger inserted through the anus to collect PF in a 1.5-ml microcentrifuge tube. PF were centrifuged at 12,000 × g for 10 min, and supernatants were collected. After a 10-fold dilution in PBS, individual and pooled PF were stored at −80°C. We obtained three seminal vesicles and epididymides from prostatic carcinoma patients aged 48 and 50 years. Epididymal fluids (EF) were diluted 10-fold in PBS. The seminal vesicles were cut into small pieces (<1 mm^3^) in PBS at a concentration of 1 g/ml to extract seminal vesicle fluids (SVF). After vortex for 2 min, the SVF were obtained by collecting supernatant after centrifugation at 12,000 × g for 10 min. The SVF were then pooled and stored at −80°C. All of the samples were used after informed consent had been given by donors and approved by the ethics committee of the PUMC.

### Viral Preparations

MuV, HSV-1, AV-5, and DeV-2 were kindly provided by laboratories of the PUMC (Beijing, China), and all viruses were amplified and titrated in Vero cells. Briefly, Vero cells (5 × 10^6^) were seeded in 100-mm culture dishes with 10 ml of DMEM supplemented with 10% FCS. After 24 h, the cells were infected with viruses at a multiplicity of infection (MOI) of 1.0. Seven days after infection, we collected the culture medium and lysed the cells by freezing in liquid nitrogen and thawing at 37°C three times. After centrifugation at 2,000 rpm for 10 min, the supernatants were collected. Viral preparations were maintained in PBS at a density of 1 × 10^9^ plaque-forming units/ml and then stored at −80°C.

### Treatments of Viruses, SP, and PF

For the pre-treatment of viruses with fluids from the male genital tracts, we incubated viruses with the defined concentration of the fluids in complete medium at 37°C for the specific durations. For heat treatment, SP and PF were heated in boiling water for 15 min. After a centrifugation at 12,000 × g for 10 min, supernatants were collected. To treat samples with enzymes, SP and PF were incubated with individual enzymes, including 300 µg/ml proteinase K (ProK) (P6565, Sigma-Aldrich, St. Louis, MO, USA), 200 U/ml DNase I (2270A, Takara Bio., Dalian, China), 200 µg/ml RNase A (GE101, TransGen Biotech, Beijing China), 200 U/ml PNGase F (G5166, Sigma), and 100 U/ml lipase (L8620, Solarbio Life Sciences Ltd. Co, Beijing, China), at 37°C for 5 h. SP and PF were then heated in boiling water for 5 min to inactivate enzymes.

### Cell Infection

Target cells were cultured in 6-well plates at 5 × 10^5^ cells/well. After 24 h, cell culture media were replaced with the pre-incubated mixtures of viruses and fluids, and the cells were incubated at 37°C for 1 h. The culture media were then replaced with virus- and fluid-free media. MuV binding to HeLa cells was determined after incubating the cells with 50 MOI MuV on ice for 1 h. After three washes with PBS, cells were collected for virus detection. For MuV entry, cells were incubated with 50 MOI MuV for 1 h at 37°C and the surface-bound MuV was removed by treating cells with 0.25% trypsin (Thermo Fisher Scientific) for 5 min. The cells were then collected for MuV detection.

### TCID_50_

Vero cells were seeded in 96-well plates at a density of 5 × 10^3^ cells/well in 100 μl of culture media. After 24 h in an incubator with 5% CO_2_ at 37°C, a serial dilution of MuV was added into plate wells. Ten days later, we calculated 50% tissue-culture effective dose (TCID_50_) values according to the Reed-Muench method.

### Cellular Viability

Cellular viability was assessed using a Cell Counting Kit-8 (CCK-8) assay kit (Dalian Meilun Biotechnology Co., Ltd., Dalian, China) according to the manufacturer’s instructions. Briefly, HeLa cells were seeded in 96-well plates at a density of 5 × 10^3^ cells/well in 100 μl culture media. At specific durations after MuV infection, 10 μl of the CCK-8 solution was added to each well. Two hours later, NADH produced by viable cells transformed colorless WST^®^-8 to orange color WST^®^-8 formazan. We measured the absorbance at 450 nm; the ratio of the absorbance value to the control (set as “1”) represented cellular viability.

### Immunofluorescence Staining

HeLa cells were cultured in 35-mm culture dishes and fixed with refrigerated 4% methanol at −20°C for 3 min. After washing them twice with PBS, the cells were permeabilized with 0.2% Triton X-100 (Zhongshan Biotechnology Co.) in PBS for 10 min and blocked with 5% normal goat serum (Zhongshan Biotechnology Co.) for 1 h at room temperature. The cells were subsequently incubated with mouse anti-MuV-NP monoclonal antibody (sc-57922) (Santa Cruz Biotechnology, Dallas, TX, USA) at 4°C in a humidified chamber for 24 h. After washing them twice with PBS, the cells were incubated with tetramethyl rhodamine isothiocyanate (TRITC)-conjugated goat anti-mouse secondary antibodies (Zhongshan Biotechnology Co.) for 1 h. Nuclei were counterstained with 4′, 6′-diamidino- 2-phenylindole (DAPI) (Zhongshan Biotechnology Co.) according to the manufacturer’s instructions. The cells were then mounted with antifade mounting medium (Zhongshan Biotechnology Co.) and observed under a fluorescence microscope BX-51 (Olympus, Tokyo, Japan).

### Real-Time Quantitative RT-PCR (qRT-PCR)

Total RNA was extracted using Trizol reagents (Thermo Fisher Scientific) according to the manufacturer’s instructions. The total RNA (1 μg) was reverse-transcribed into cDNA in a 20-μl reaction mixture containing 2.5 μM of random hexamers, 2 μM of deoxynucleotide triphosphates, and 200 units of Moloney murine leukemia virus reverse transcriptase (Promega, Madison, WI, USA). We performed PCR in a 20-μl reaction mixture containing 0.2 μl of cDNA, 0.5 μM of forward and reverse primers, and 10 μl of Power SYBR Green PCR Master Mix (Applied Biosystems, Foster City, CA, USA) on an ABI PRISM 7300 real-time cycler (Applied Biosystems). Relative mRNA levels were determined using the 2^−ΔΔCt^ method, as described in the Applied Biosystems User Bulletin No. 2 (P/N 4303859). The primer sequences for PCR are listed in **Table 1**.

**Table 1:** Primers used for real-time qRT-PCR.

Target genes	Forward (5’ → 3’)	Reverse (5’ → 3’)
MuV-NP	TCAGATCAATCGCATCGGGG	CTTGCGACTGTGCGTTTTGA
HSV-1-UL49	GGTGTTCGTCGTCTTCGGAT	CTTCAGGTATGGCGAGTCCC
AV-5-Hexon	GGTGGCCATTACCTTTGACTCTTC	CCACCTGTTGGTAGTCCTTGTATTTAGTATCATC
DeV-2-NS1	AATCCGCTCAGTAACAAG	GTTTCGGGACCATCAATA
*ACTB*	CATGTACGTTGCTATCCAGGC	CTCCTTAATGTCACGCACGAT
*Actb*	GAAATCGTGCGTGACATCAAAG	TGTAGTTTCATGGATGCCACAG

NP, nuclear protein; UL, unique long gene; NS, non-structural protein.

### Western Blot Analysis

Cells were lysed in a lysis buffer containing a protease inhibitor cocktail (Sigma-Aldrich), and the protein concentrations were determined using a bicinchoninic acid protein assay kit (Pierce Biotechnology, Rockford, IL, USA). Proteins (20 μg/well) were separated using 10% SDS-PAGE and then electro-transferred onto polyvinylidene fluoride membranes (Millipore, Bedford, MA, USA). The membranes were blocked with Tris-buffered saline (pH 7.4) containing 5% non-fat milk for 1 h at room temperature and then incubated with mouse anti-MuV-NP (ab9880) (Abcam, Cambridge, UK) or anti-β-Actin (66009-1-lg) (Sigma-Aldric) mAbs for 24 h at 4°C. After washing them twice with Tris-buffered saline containing 0.1% Tween-20, the membranes were incubated with HRP-conjugated secondary antibodies for 1 h at room temperature. Protein bands were visualized with an Enhanced Chemiluminescence Detection Kit (Zhongshan Biotechnology Co.).

### Ultrafiltration of SP and PF

SP and PF were separated to four fractions (<3 kDa, >3 kDa, <100 kDa, >100 kDa) by ultrafiltration using Amicon® Ultra-0.5 Centrifugal Filter Devices (Merck Millipore Co., Darmstadt, Germany) according to manufacturer’s instructions. Briefly, 500 µl of sample was added to filter device and capped it. The device was spun at 12,000 × g for 30 min. The filtrate was collected. The retentate was washed twice with PBS by repeating centrifugation. The filter device was placed upside down in a 1.5 ml micro-centrifuge tube and spun at 1,000 × g for 2 min to obtain the retentate. The retentate was diluted to a final volume of 500 µl.

### Statistical Analysis

Statistical difference between two groups was determined using Student’s *t* test. A one-way ANOVA with Bonferroni’s (selected pairs) *post-hoc* test was used for multiple comparisons. We performed calculations using SPSS version 13.0 (SPSS Inc., Chicago, IL, USA). Data were presented as the means ± SEM of at least three experiments. A *P*-value <0.05 was considered to be statistically significant.

## Results

### MuV Infection of HeLa Cells

**Figure 1:**
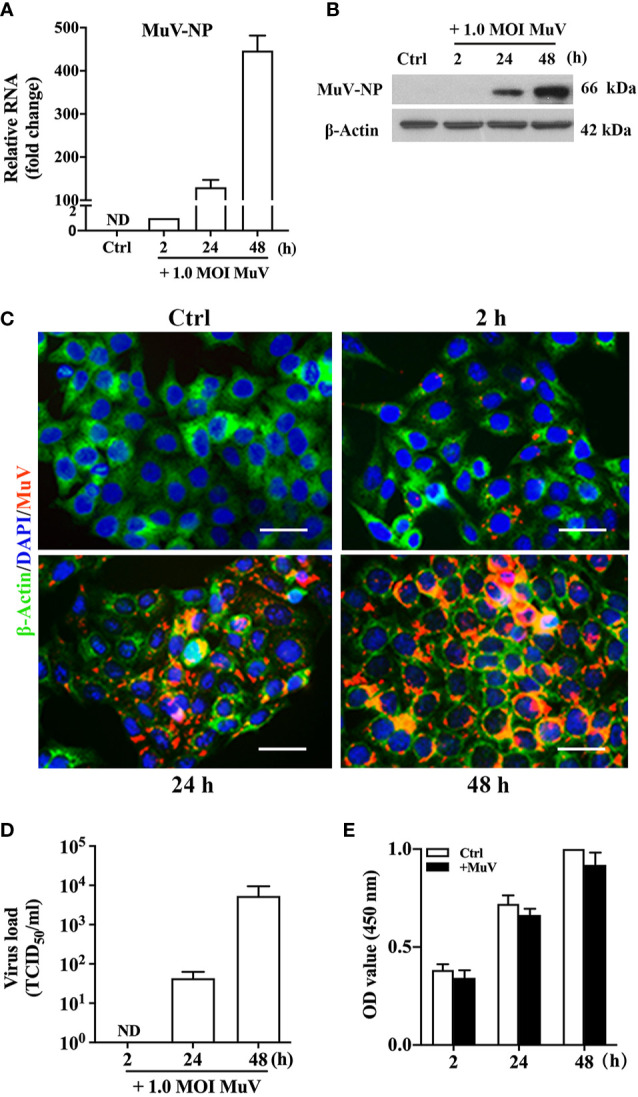
MuV replication in HeLa cells. **(A)** Time-dependent MuV replication. HeLa cells were infected with MuV at a MOI of 1.0, and cells without MuV infection served as controls (Ctrl). Total RNA was extracted at specific times after infection, and the relative RNA levels of MuV-NP were analyzed using real-time qRT-PCR. **(B)** Protein levels of MuV-NP. HeLa cells were infected with MuV as described in **(A)**. At the indicated time-points after infection, MuV-NP protein levels in the cellular lysates were determined using Western blot analysis. **(C)** Intracellular MuV-NP. HeLa cells were infected with MuV as described in **(A)**. We determined intracellular distribution of MuV-NP (red) using immunofluorescence staining. The cytoplasm and nuclei were localized by immunofluorescence staining for β-Actin (green) and DAPI (blue). **(D)** Viral loads. HeLa cells were infected with 1.0 MOI MuV for the indicated periods of time. MuV loads in culture medium were determined using a TCID_50_ assay. **(E)** Cell viability. After infection with MuV at a MOI of 1.0 for the indicated durations, the HeLa cell viability was determined using the CCK-8 assay. Images represent at least three independent experiments. Data are presented as means ± SEM of three separate experiments. Scale bar = 20 μm. ND, not detectable.

To evaluate MuV infection and replication *in vitro*, human cervical epithelium cancer-derived HeLa cells were used as host cells. Real-time qRT-PCR results showed that MuV efficiently infected HeLa cells and replicated within them in a time-dependent manner (**Figure 1A**). The RNA levels of MuV nuclear protein (MuV-NP) gene were dramatically increased within cells at 24 and 48 h after infection with MuV at a MOI of 1.0. Accordingly, the protein levels of MuV-NP were increased in HeLa cells in a time-dependent manner after infection (**Figure 1B**). MuV-NP was further confirmed using immunofluorescence staining, which showed much greater fluorescent densities in cells at 24 and 48 h after infection (**Figure 1C**, lower panels). TCID_50_ assay results also showed that viral loads in the HeLa cell culture medium significantly increased in a time-dependent manner (**Figure 1D**). MuV infection did not significantly affect cellular viability (**Figure 1E**).

### Inhibition of MuV Infection by SP

**Figure 2:**
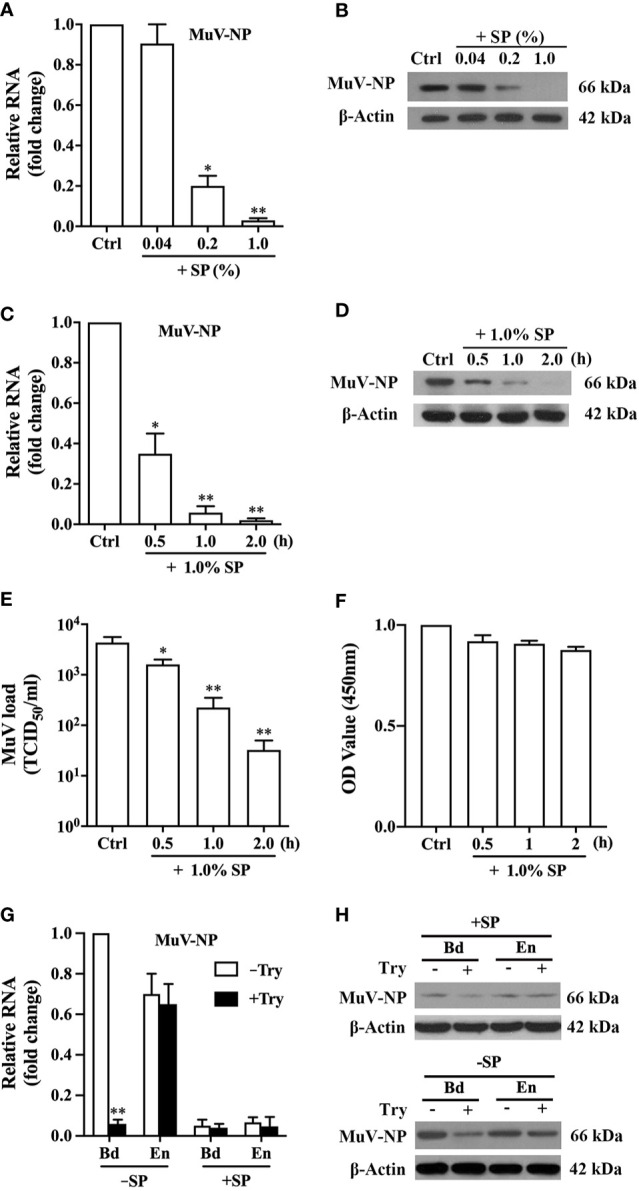
Inhibition of MuV infection by SP. **(A)** Dose-dependent SP inhibition of MuV infection. MuV was pre-incubated with the indicated doses of SP for 2 h, and MuV without SP treatment served as controls (Ctrl). HeLa cells were infected with MuV. Total RNA was extracted at 48 h after MuV infection, and MuV-NP RNA levels were determined using real-time qRT-PCR. **(B)** HeLa cells were treated as described in **(A)**. The protein levels of MuV-NP were determined using Western blot analysis. **(C, D)** Time-dependent SP inhibition of MuV infection. MuV was pre-incubated with 1.0% SP for the specific durations. HeLa cells were infected with MuV for 48 h. MuV-NP RNA **(C)** and protein **(D)** levels were determined with real-time qRT-PCR and a Western blot analysis, respectively. **(E)** MuV loads in culture medium. MuV was treated, and HeLa cells were infected as described in **(C)**. Culture supernatants were collected at 48 h after MuV infection, and MuV loads in culture supernatants were determined using the TCID_50_ assay. **(F)** Cell viability. HeLa cells were treated as described in **(C)**, and the cell viability was assessed using the CCK-8 assay. **(G, H)** MuV binding (Bd) and entry (En). MuV was pre-incubated in the absence (−SP) or the presence (+SP) of 1.0% SP for 2 h. HeLa cells were inoculated with 50 MOI of MuV on ice for 1 h to detect MuV binding to cells. HeLa cells were inoculated with 50 MOI of MuV at 37°C for 1 h to examine MuV entry into cells. MuV bound to cells was removed by treatment with 0.25% trypsin (+Try) for 5 min. MuV-NP RNA **(G)** and protein **(H)** levels were determined using real-time qRT-PCR and Western blot analysis, respectively. Images represent at least three independent experiments. Data are presented as means ± SEM of three experiments. *P < 0.05, **P < 0.01.

To analyze the effect of SP on MuV infection, MuV-NP level was examined in HeLa cells after inoculation with a pre-incubation mixture of SP and MuV. A 2-h pre-incubation of 1.0 MOI MuV with SP at 37°C significantly attenuated MuV-NP RNA levels in HeLa cells in a dose-dependent manner at 48 h after infection (**Figure 2A**). In addition, Western blot analysis showed that MuV-NP protein levels were reduced by the presence of SP (**Figure 2B**). A 2-h pre-incubation of MuV with 1.0% SP dramatically inhibited MuV infection. The inhibitory efficiency of 1.0% SP on MuV infection depended upon the duration of the pre-incubation of MuV with SP; that is, the 2-h pre-incubation of 1.0 MOI MuV with 1.0% SP markedly reduced MuV-NP RNA (**Figure 2C**) and protein (**Figure 2D**) levels. Accordingly, SP significantly reduced MuV loads in the culture medium in a pre-incubation time-dependent manner 48 h after infection (**Figure 2E**). The presence of 1.0% SP for up to 2 h did not impair HeLa cell viability (**Figure 2F**). These results suggested that SP contains an antiviral factor that potently inhibits MuV infection. However, the presence of 1.0% SP for long terms could significantly reduce cell viability (**Figure S1**). We therefore incubated HeLa cells with SP for 1 h to avoid the cytotoxic effect in the present study.

To confirm whether SP acted on viruses or target cells, we firstly treated HeLa cells with 1.0% SP for 1 h, and the cells were then infected with 1.0 MOI MuV. The pre-treatment of HeLa cells with SP did not affect MuV infection (**Figure S2**). Further, we compared MuV infection after the pre-incubation of MuV with SP at 37, 4, or 45°C for 2 h to determine the association between the antiviral effect of SP and temperature. MuV infection was significantly inhibited by the pre-incubation at 4°C while the inhibition was evidently reduced in comparison with the pre-incubation at 37°C (**Figure S3**). However, MuV infection was completely abolished at 45°C in both the presence and absence of SP.

To determine which step of the viral infection cascade was inhibited by SP, we detected MuV binding and entry. MuV efficiently bound (Bd) with and entered (En) into HeLa cells in the absence of SP (**Figure 2G**). Treatment of cells with 0.25% trypsin for 5 min significantly removed bound MuV-NP RNA, but did not affect the entered MuV-NP RNA level. The 2-h pre-incubation of MuV with SP almost completely blocked MuV binding and entry to cells because Bd and En MuV-NP RNA levels were extremely low. Western blotting results confirmed that the pre-incubation of MuV with SP markedly reduced Bd and En MuV-NP protein levels (**Figure 2H**, upper panel). Trypsin evidently reduced Bd MuV-NP but did not affect En MuV-NP levels (**Figure 2H**, lower panel). The diminished MuV entry in the presence of SP was most likely due to the impairment of virus binding as it is essential for virus entry.

### Characterization of Seminal Antiviral Factor

**Figure 3:**
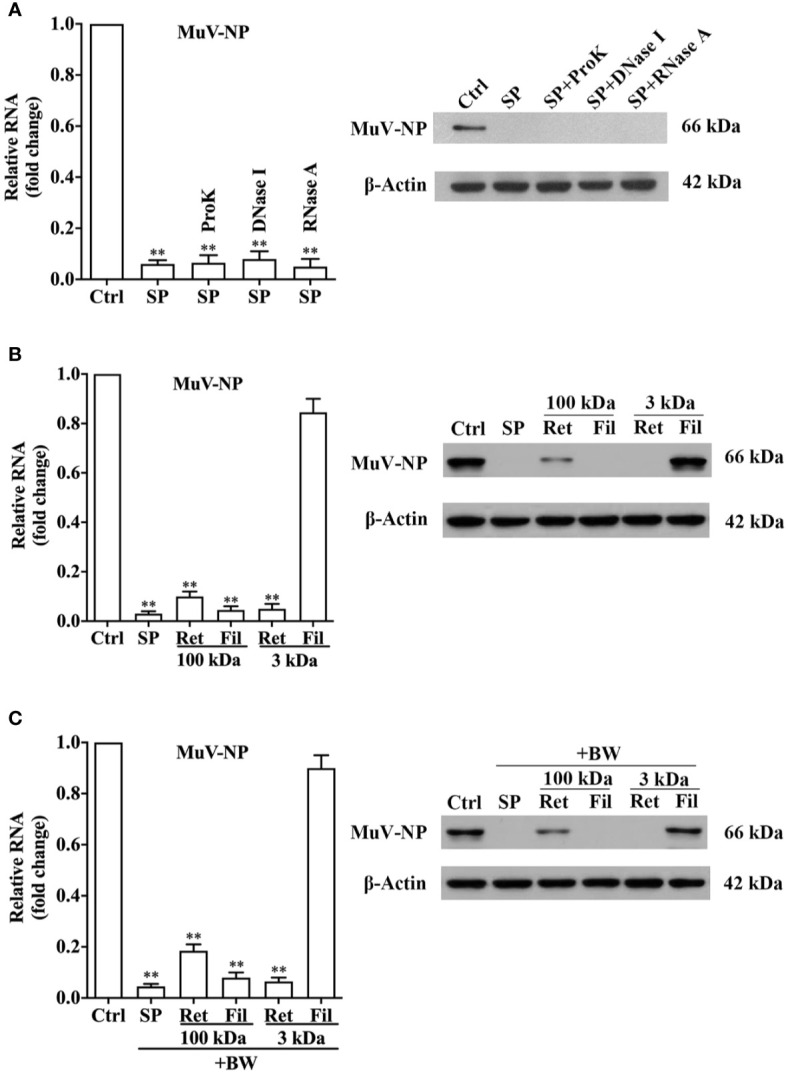
Characterization of antiviral component. **(A)** Physical properties of the antiviral component. SP was treated with proteinase K (ProK), DNase I, or RNase A at 37°C for 5 h. MuV was incubated with SP for 2 h, and MuV without treatment served as the control (Ctrl). HeLa cells were infected with 1.0 MOI of MuV. At 48 h post-infection, MuV-NP RNA (left panel) and protein (right panel) levels were determined using real-time qRT-PCR and a Western blot, respectively. **(B)** Size analysis of the antiviral component. SP was filtered through 100 kDa and 3 kDa filters. Filtrate (Fil) and retentate (Ret) fractions were collected. MuV was incubated with fractions for 2 h, and HeLa cells were infected with MuV. MuV-NP RNA (left panel) and protein (right panel) levels were determined. **(C)** Heat resistance of the antiviral component. The Fil and Ret fractions of ultrafiltration were treated with boiling water (BW) for 10 min, and then incubated with MuV for 2 h. MuV-NP RNA (left) and protein (right) levels were determined. Images represent at least three independent experiments. Data are presented as means ± SEM of three experiments, **P < 0.01.

To characterize the properties of the antiviral factor, SP was treated with protein- and nucleic acid-degrading enzymes for 5 h at 37°C. Treatments of SP with either ProK, DNase I, or RNase A did not alter MuV-NP RNA (**Figure 3A**, left panel) or protein (right panel) levels in HeLa cells 48 h after infection. ProK, DNase I, and RNase A efficiently digested seminal proteins, isolated DNA and RNA from HeLa cells, respectively (**Figure S4**). Moreover, the treatment of SP with PNGase F and lipase retained the SP inhibition of MuV infection (**Figure S5**). In controls, the treatment of MuV with each enzyme alone without SP did not affect MuV infection (**Figure S6**). The results suggest that proteins, nucleic acids, glycosylated molecules, and lipids were not responsible for the antiviral effect of SP.

The molecular size of the antiviral factor was assessed by SP filtration through 100-kDa and 3-kDa filters. A 2-h pre-incubation of MuV with the retentate (Ret) of both filters dramatically reduced MuV-NP RNA (**Figure 3B**, left panel) and protein (right panel) levels. The SP filtrate (Fil) of 100-kDa filter also evidently reduced MuV-NP levels, whereas the filtrate of 3-kDa filter did not affect MuV infection. Moreover, the treatment of the retentate of two filters with boiling water did not markedly reduced the inhibitory effects on MuV infection (**Figure 3C**). Notably, SDS-PAGE results showed that the retentate of 100-kDa filter contained abundant <100 kDa molecules, whereas the filtrate of 100-kDa filter did not contain proteins of >100-kDa (**Figure S7**), which indicate that the ultrafiltration through 100-kDa filter did not efficiently separated >100-kDa molecules from smaller molecules. These results suggest that the antiviral component exists in >3kDa fraction, but not in <3 kDa fraction.

### Inhibition of MuV Infection by Prostate Fluids

**Figure 4:**
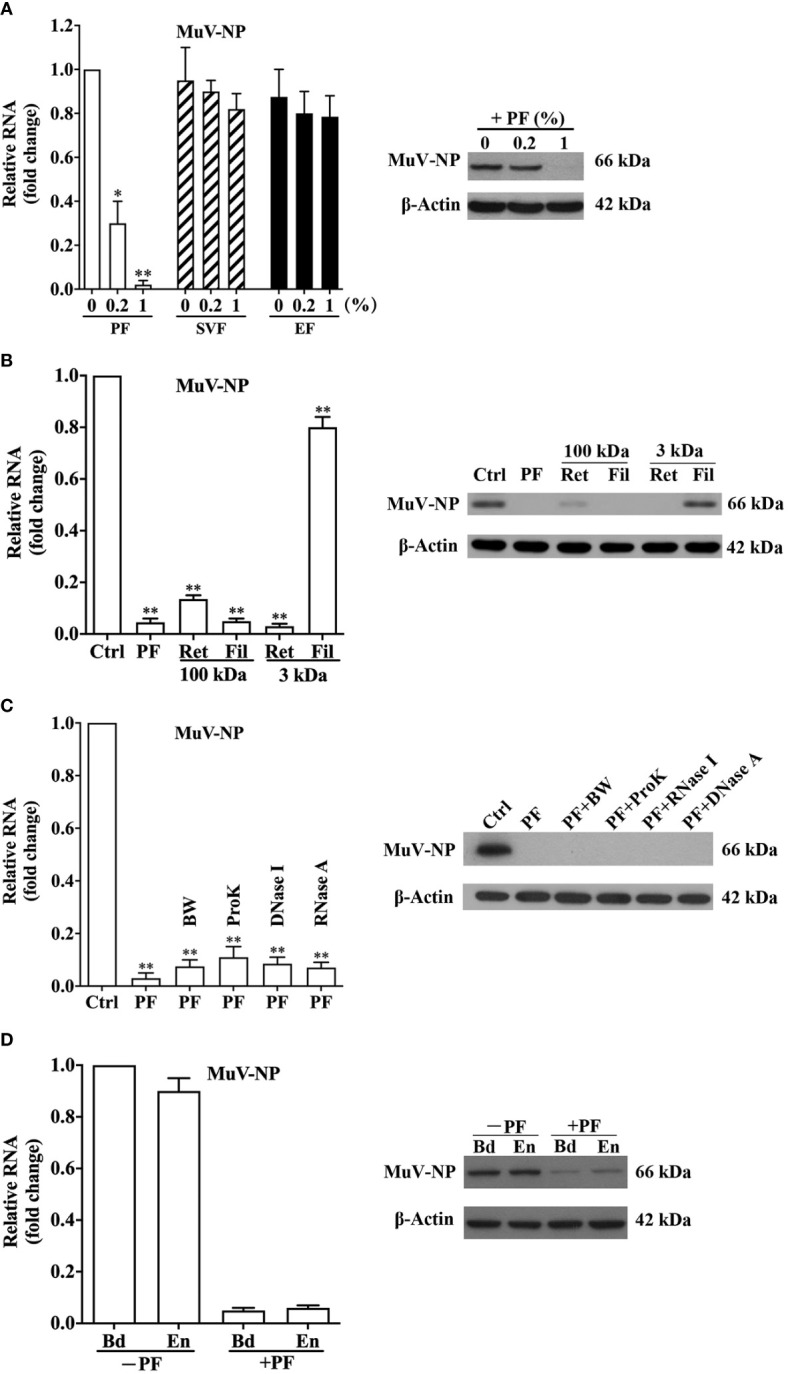
Antiviral effect of male genital tract fluids. **(A)** Antiviral effect of prostatic fluids (PF), seminal vesicle fluids (SVF), and epididymal fluids (EF). MuV was incubated with the indicated doses of PF, SVF, or EF at 37°C for 2 h, and HeLa cells were infected with 1.0 MOI of MuV. At 48 h after infection, MuV-NP RNA levels (left panel) were determined using real-time qRT-PCR. The inhibition of PF on MuV-NP protein level (right panel) was analyzed by a Western blot. **(B)** Antiviral effect of PF fractions. PF were filtered through a 3-kDa filter, and MuV was incubated with either 1% filtrate (Fil) or 1% retentate (Ret) at 37°C for 2 h. MuV without treatment served as the control (Ctrl). HeLa cells were infected with 1.0 MOI of MuV. MuV-NP RNA (left panel) and protein (right panel) levels were determined 48 h after infection. **(C)** Properties of the antiviral factor in PF. PF were treated in boiling water (BW) for 5 min, or incubated with Proteinase K (ProK), DNase I, or RNase A at 37°C for 5 h, followed by heating inactivation in BW for 5 min. MuV was incubated with 1% PF for 2 h, and HeLa cells were infected with 1.0 MOI of MuV. MuV-NP RNA and protein levels were determined 48 h after infection. **(D)** MuV binding (Bd) and entry (En). MuV was pre-incubated with 1% PF (+PF) or without PF (-PF) at 37°C for 2 h. HeLa cells were inoculated for 1 h with 50 MOI MuV on ice for binding or at 37°C for entry. MuV-NP RNA (left panel) and protein (right panel) levels were determined. Images represent at least three independent experiments. Data are presented as means ± SEM of three experiments. *P < 0.05, **P < 0.01.

Given that SP is mostly produced by the prostate, seminal vesicle, and epididymis, we examined the antiviral effect of the prostatic fluids (PF), seminal vesicle fluids (SVF), and epididymal fluids (EF). The pre-incubation of MuV with PF at doses of 0.2 and 1% significantly reduced MuV-NP RNA levels in HeLa cells 48 h after infection, whereas SVF and EF did not affect MuV-NP RNA levels (**Figure 4A**, left panel). Accordingly, PF markedly reduced MuV-NP protein level at a dose of 1% (**Figure 4A**, right panel). The retentate of PF through a 3-kDa filter dramatically reduced MuV-NP RNA (**Figure 4B**, left panel) and protein (right panel) levels, although the filtrate did not affect MuV-NP levels. Both the retentate and filtrate through a 100-kDa filter significantly inhibited MuV infection. Treatment of PF with boiling water (BW), proteinase K (ProK), DNase I, or RNase A did not affect MuV-NP RNA (**Figure 4C**, left panel) and protein levels (right panel). The 2-h pre-incubation of MuV with PF dramatically diminished bound and entered MuV-NP RNA (**Figure 4D**, left panel) and protein (right panel) levels. These observations are similar to the those for SP, suggesting that the antiviral component in SP is most likely produced by the prostate gland.

### Individual Variations in Antiviral Effect

**Figure 5:**
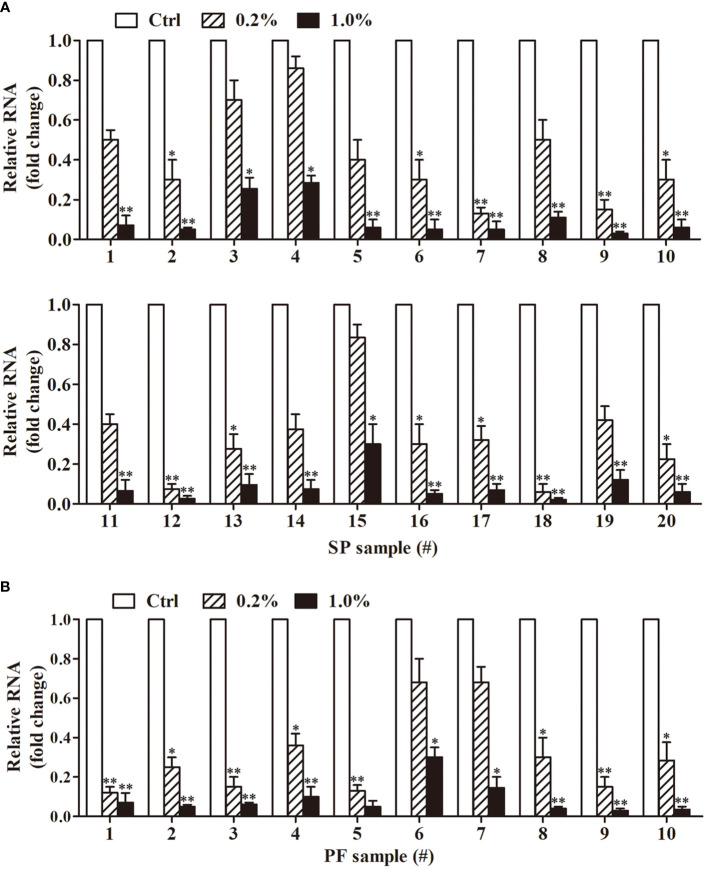
Antiviral effect of SP and PF from individual donors. **(A)** Antiviral effect of SP. SP samples were collected from 20 individual donors. MuV was incubated with 0.2 or 1.0% individual SP samples for 2 h. MuV without treatment served as the control (Ctrl). HeLa cells were infected with 1.0 MOI of MuV. Total RNA was extracted from cells 48 h after MuV infection. MuV-NP RNA levels were determined using real-time qRT-PCR. **(B)** Antiviral effect of individual PF samples. Individual PF samples were collected from 10 donors. MuV was incubated with individual PF, and HeLa cells were infected as described in **(A)**. MuV-NP RNA levels were determined 48 h after infection. Data are presented as means ± SEM of three experiments. *P < 0.05, **P < 0.01.

To compare the antiviral activities of SP and PF from individual donors, we examined the antiviral effect of 20 SP and 10 PF samples. All 20 of the SP samples at a dose of 1% dramatically reduced the MuV-NP RNA level in HeLa cells 48 h after MuV infection (**Figure 5A**). In contrast, 0.2% SP exhibited great variation in inhibiting MuV infection. Although the 0.2% SP treatment of samples 7, 9, 12, and 18 greatly reduced MuV-NP RNA levels, other SP samples did not significantly inhibit MuV infection at a dose of 0.2%. Similarly, 1% of all PF samples substantially decreased MuV-NP RNA levels (**Figure 5B**). Although 8 of 10 PF samples significantly reduced MuV-NP RNA levels at the dose of 0.2%, samples 6 and 7 did not significantly reduce MuV-NP RNA at this dose (**Figure 5B**). These results indicated that the antiviral effect is a general property of human SP and PF with variation in individual donors.

### Pan-Antiviral Effect of the Antiviral Factor

**Figure 6:**
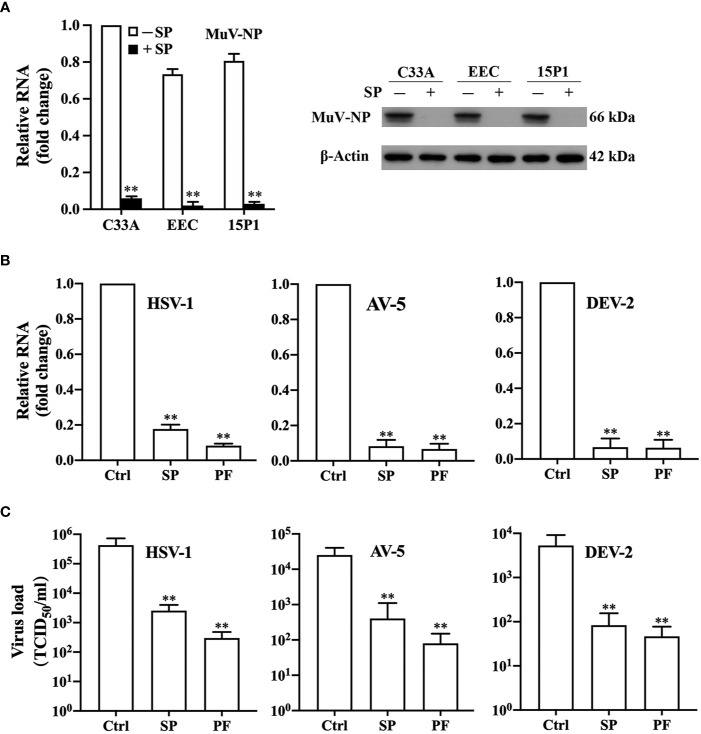
Pan-antiviral effects of antiviral factor. **(A)** SP effect on MuV infection of different cell types. MuV was incubated with (+) 1% SP or without (−) SP at 37°C for 2 h. Human cervical squamous carcinoma cell line C33A, mouse primary epididymal epithelial cell (EEC), and mouse Sertoli cell line 15P1 were infected with 1.0 MOI MuV. MuV-NP RNA (left panel) and protein (right panel) levels were determined at 48 h after MuV infection. **(B, C)** Antiviral effect of SP against different viruses. **(B)** Viral RNA. Herpes simplex virus 1 (HSV-1), adenovirus 5 (AV-5), and dengue virus 2 (DeV-2) were incubated with 1% SP or PF for 2 h. Viruses without treatment served as the controls (Ctrl). HeLa cells were infected with each virus at a MOI of 1.0. RNA levels of HSV-1 (left panel), AV-5 (middle panel), and DeV-2 (right panel) were determined using real-time qRT-PCR 48 h after infection. **(C)** Viral loads in culture medium. Viruses were treated, and HeLa cells were infected with each virus, as described in **(B)**. Culture medium was collected 48 h after infection. Viral loads of HSV-1 (left panel), AV-5 (middle panel), and DeV-2 (right panel) in the medium were determined using TCID_50_. Data are presented as means ± SEM of three experiments. **P < 0.01.

To determine whether the SP inhibition of MuV infection is a cell line-specific effect, we performed the experiments on three other cell types, including C33A, EEC, and 15P1. Similar to the observations in HeLa cells, the pre-incubation of MuV with 1.0% SP remarkably reduced MuV infection in all these cells (**Figure 6A**). To examine whether the antiviral factor specifically inhibits MuV infection or generally inhibits infection by other viruses, we analyzed the effect of SP and PF on the infection of HeLa cells by HSV-1, AV-5, and DeV-2. The pre-incubation of these viruses with 1.0% SP or PF dramatically reduced the RNA levels of HSV-1 (**Figure 6B**, left panel), AV-5 (middle panel), and DeV-2 (right panel) in HeLa cells 48 h after infection. Similarly, SP and PF significantly attenuated loads of HSV-1 (**Figure 6C**, left panel), AV-5 (middle panel), and DeV-2 (right panel) in the culture medium. These results indicated that the antiviral factor in SP and PF exerts a pan-antiviral activity.

## Discussion

The presence of viruses in semen is a risk for sexual spread of pathogens. Semen is not only a passive vector for viral spread, but also impacts viral transmission. The facilitative and inhibitory effects of semen on HIV-1 transmission have been documented (32). In the present study, we examined the antiviral effect of SP. We demonstrated that human SP decidedly inhibited MuV, HSV-1, AV-5, and DeV-2 infection, which suggest a pan-antiviral effect of SP.

The male reproductive system is a potential refuge for harboring viruses due to its immunosuppressive environment for protecting immunogenic germ cells from autoimmune responses (33). The mammalian testis, in particular, is a remarkably immunoprivileged organ wherein viruses can escape from immune surveillance and impair testicular function (34, 35). The antiviral effect of SP constitutes an important strategy for restricting the sexual transmission of viruses. MuV manifests a tropism for the testis and frequently induces orchitis (21). While natural MuV-induced diseases have been only observed in human beings, MuV can infect different species (29). Accordingly, the present study showed that several cell types from the human female genital tract and the mouse male genital tract were efficiently infected *in vitro* by MuV, and that SP potently inhibited MuV infection of the target cells. These results suggest that the antiviral effect of SP is a general property independent target cells.

The effect of semen on HIV-1infection has been intensively studied because sexual transmission is a major route of viral spread. Semen may inhibit HIV-1 infection through distinct mechanisms. Early studies showed that SP abrogated HIV-1 infection through gp17 glycoprotein, cationic polypeptides, DC-SIGN (7, 36). Several other studies demonstrated that exosomes in human semen were involved in the inhibition of HIV-1 infection (8–10). Recent studies showed that seminal exosomes and salivary extracellular vesicles inhibited ZIKV infection through the impairment of viral attachment to target cells by acting on cells (12, 37). These mechanisms could not explain SP inhibition of MuV infection in the present study. We demonstrated that SP acted on MuV and did not target host cells. Moreover, the fraction of <100 kDa potently inhibited MuV infection, which did not support the involvement of exosomes. While the retentate of 100 kDa filter also significantly inhibited MuV infection, we could not conclude that it was indeed the effect of a >100 kDa component because this fraction contained abundant <100 kDa components. We also demonstrated that heating at boiling water did not affect the inhibitory effect of SP on MuV infection, and that neither proteins nor glycosylated molecules were involved in the SP inhibition of MuV infection, which corresponded the observations in the inhibition of semen on ZIKV infection (12). We further excluded the involvement of nucleic acids and lipids because the treatments of SP with respective enzymes did not restrain the inhibition of MuV infection. After the exclusion of these macromolecules, it is hard to image what the antiviral factor is. We may speculate that a small molecule that naturally binds to the macromolecules poses the virucidal effect of SP. Since the antiviral factor likely produced by the prostate, the analysis on the products of the prostatic cells *in vitro* would aid in the identification of the antiviral factor. The antiviral component in SP potently inhibited viral infection because 1% SP profoundly inhibited viral infection. The antiviral effect of SP is an interesting issue that is worthy of further investigation.

To understand the mechanisms by which SP inhibited MuV infection, we explored the targets acted upon by the antiviral factor. A time-dependent pre-incubation of MuV with SP was required for the antiviral effect. By contrast, the pre-treatment of HeLa cells with SP did not alter MuV infection. These results suggest that the antiviral factor acted on viruses and did not act on target cells. These results differ the observation that semen inhibited ZIKV infection by acting on target cells *via* extracellular vesicles (12), suggesting that semen possesses different antiviral components against viral infection through distinct mechanisms. Although SP is highly toxic to cell culture *in vitro*, we used a low dose of 1% SP and treated cells for a short time of 1 h to reduce the cytotoxicity. We provided evidence that the treatment of HeLa cells with 1% SP up to 2 h did not significantly impair cell viability and did not inhibit MuV infection, which excluded involvement of the cytotoxic effect in the SP inhibition of MuV infection.

SP is principally produced by accessory sex glands, including the seminal vesicle and prostate glands, which contribute to approximately 70 and 25% of SP, respectively (38, 39). The epididymis also produces ~5% of SP. An antiviral effect was only observed with PF, whereas SVF and EF did not inhibit MuV infection, suggesting that the antiviral component in SP was produced by the prostate. Numerous viruses have been found and persist long-term in the testis because of the testicular immunoprivileged status (3). Viruses may be shed from the testis into semen. In addition to the mucosal barrier and viral tropism, the production of an antiviral factor by the prostate gland should restrict the overall sexual transmission of viruses.

In contrast to the antiviral effects of semen, several previous studies focused on the role of semen in facilitating HIV-1 infection (32). These studies demonstrated that seminal amyloid fibrils enhanced HIV-1 infection by facilitating the attachment of HIV-1 to targets cells (14). This enhancement was also reported for HCMV infection (16). Notably, a recent study demonstrated that SP inhibited HCMV infection (13). Our present study provided substantial evidence that SP inhibited MuV infection of different cell types, including human cervical carcinoma cell lines, mouse primary epididymal epithelial cells, and a mouse Sertoli cell line. We showed that the antiviral component produced by the prostate and acted directly on the virus. Moreover, we found that SP also inhibited HSV-1, AD-5, and DeV-2 infection. The discrepancies in the results from these different studies have yet to be resolved. We realize that seminal amyloid fibrils always promote infection of different viruses, whereas the effect of whole SP on viral infection remain controversial. Considering that different virus strains and target cells were used in the previous studies, the effect of SP on viral infection should depend on the virus strains and host cells through different mechanisms. The virus- and host cell-specific antiviral effect of SP is an interesting issue for future study. Notably, the infected or inflammatory status of the male genital tract may impact HIV-1 infection (19). Since MuV has a tropism for the testis and epididymis and induces a severe inflammation in these organs, the potential modification of SP due to inflammation in the male genital tract may impact the antiviral effect of SP, which should be considered and worthy of clarification.

In summary, the immunoprivileged status of the male reproductive tract provides a sanctuary for viruses to escape immune surveillance. These viruses may shed into semen, thereby transmitting *via* sexual activity. It is important for the male reproductive system to adopt mechanisms that inhibit viral infection for restricting sexual transmission of the viruses. In the present study, we characterized a pan-antiviral property of SP and PF that potently inhibited infection of various types of cells by different viruses. Our results provided novel insights into the antiviral effect of SP and we believe that the isolation of specific antiviral factors from semen may benefit antiviral therapy.

## Data Availability Statement

The original contributions presented in the study are included in the article/**Supplementary Material**. Further inquiries can be directed to the corresponding author.

## Ethics Statement

The studies involving human participants were reviewed and approved by the Institutional Review Board of Institute of Basic Medical Sciences, Chinese Academy of Medical Sciences. The patients/participants provided their written informed consent to participate in this study. Written informed consent was obtained from the individual(s) for the publication of any potentially identifiable images or data included in this article.

## Author Contributions

RC, WZ, MG, and DH designed the experiments. RC, WZ, MG, FW, HW, WLi, YG, BL, SC, WLu, XY, and AL performed the experiments. RC, WZ, MG, FW, RH, YC, and DH analyzed the data. DH, RC, WZ, and MG wrote the paper, with the other authors providing editorial comments. All authors contributed to the article and approved the submitted version.

## Funding

This work was supported by grants from the National Key R&D program of China (Nos. 2018YFC1003900 and 2016YFA0101001), the National Natural Science Foundation of China (No. 82071633), CAMS Initiative for Innovative Medicine (Nos. 2017-I2M-B&R-06 and 2017-I2M-3-007).

## Conflict of Interest

The authors declare that the research was conducted in the absence of any commercial or financial relationships that could be construed as a potential conflict of interest.

## Supplementary Material

Supplementary Figure 1 Long-term effect of SP on cell viability. HeLa cells were incubated with 1.0% SP for the specific durations. Culture media were replaced with fresh complete media without SP. The cell viability was assessed using the CCK-8 assay 48 h after the medium replacements. Data are presented as means ± SEM of three experiments. **P* < 0.05, ***P* < 0.01.

Supplementary Figure 2 SP effect on cells and MuV. HeLa cells were pre-incubated with 1% SP (C+SP) for 1 h and then transfected with 1.0 MOI MuV. MuV was pre-incubated with 1% SP (MuV+SP) and then transfected HeLa for 1 h. HeLa cells were transfected with MuV without SP served as the control (Ctrl). At 48 h after infection, MuV-NP RNA (left panel) and protein (right panel) levels were determined using real-time qRT-PCR and Western blot, respectively. Data are the means ± SEM of three experiments and images represent at least three independent experiments. ***P* < 0.01.

Supplementary Figure 3 Temperature-dependent antiviral effect of SP. MuV was incubated with 1% SP (+) or without SP (−) for 2 h at 4, 37, or 45°C. HeLa cells were infected with 1.0 MOI MuV. MuV-NP RNA and protein levels were determined using real-time qRT-PCR and Western blot. Data are the means ± SEM of three experiments. Images represent three independent experiments. **P* < 0.05, ***P* < 0.01.

Supplementary Figure 4 Efficiency of enzymes. **(A)** Proteinase K (ProK). Seminal plasma (SP) was incubated with 300 μg/ml (+) or without (−) ProK at 37°C for 5 h and then heated in boiling water for 5 min. Each sample (2 μl/well) was loaded for SDS-PAGE with 10% polyacrylamide gel. Proteins were visualized after staining with Coomassie blue and following by decoloration. Molecular sizes were referred by protein markers (M). **(B, C)** Nucleic acid enzymes. Genomic DNA and total RNA were isolated from HeLa cells. DNA **(B)** or RNA **(C)** were treated with 200 U/ml DNase I or 200 μg/ml RNase A at 37°C for 5 h. DNA and RNA were determined after electrophoresis in 1% agarose. Images are the representatives of at least three independent experiments.

Supplementary Figure 5 PNGase F and lipase treatments. Seminal plasma (SP) was treated with 200 U/ml PNGase F or 100 U/ml lipase at 37°C for 5 h. MuV was incubated with SP for 2 h. MuV without SP served as the control (Ctrl). HeLa cells were infected with 1.0 MOI MuV. MuV-NP RNA (left panel) and protein (right panel) levels were determined at 48 h after infection using real-time qRT-PCR and Western blot, respectively. Images represent three experiments. Data are the means ± SEM of three experiments. ***P* < 0.01.

Supplementary Figure 6 Effect of enzymes on MuV infection. **(A)** Proteinase K (ProK), DNase I, and RNase A were heated in boiling water for 5 min. MuV was incubated with 300 μg/ml Pro K, 200 U/ml DNase I, and 200 μg/ml RNase A for 5 h. MuV without enzymes served as the control (Ctrl). HeLa cells were infected with 1.0 MOI MuV, and MuV-NP RNA (left panel) and protein (right panel) were determined at 48 h after MuV infection. **(B)** PNGase F and lipase were heated in boiling water for 5 min. MuV was incubated with 200 U/ml PNGase F or 100 U/ml lipase for 5 h. HeLa cells were infected and MuV-NP was determined as described in **(A)**. Data are the means ± SEM of three experiments, and images are the representatives of three experiments.

Supplementary Figure 7 Fractions of seminal plasma (SP). SP was separated to four fractions by ultrafiltration using 100 kDa and 3 kDa filter devices. The retentates (Ret) and filtrates (Fil) of the two filters were subjected to SDS-PAGE with 10% polyacrylamide gel. The protein components were visualized after Coomassie blue staining and decoloration. Protein sizes were referred by molecular markers (M). Images represent three experiments.
