# Supplementary figures and images for: Characterization of an Antiviral Component in Human Seminal Plasma
Source: Front Immunol. 2021 Feb 19;12:580454. doi: 10.3389/fimmu.2021.580454 (PMC7933687; doi:10.3389/fimmu.2021.580454)

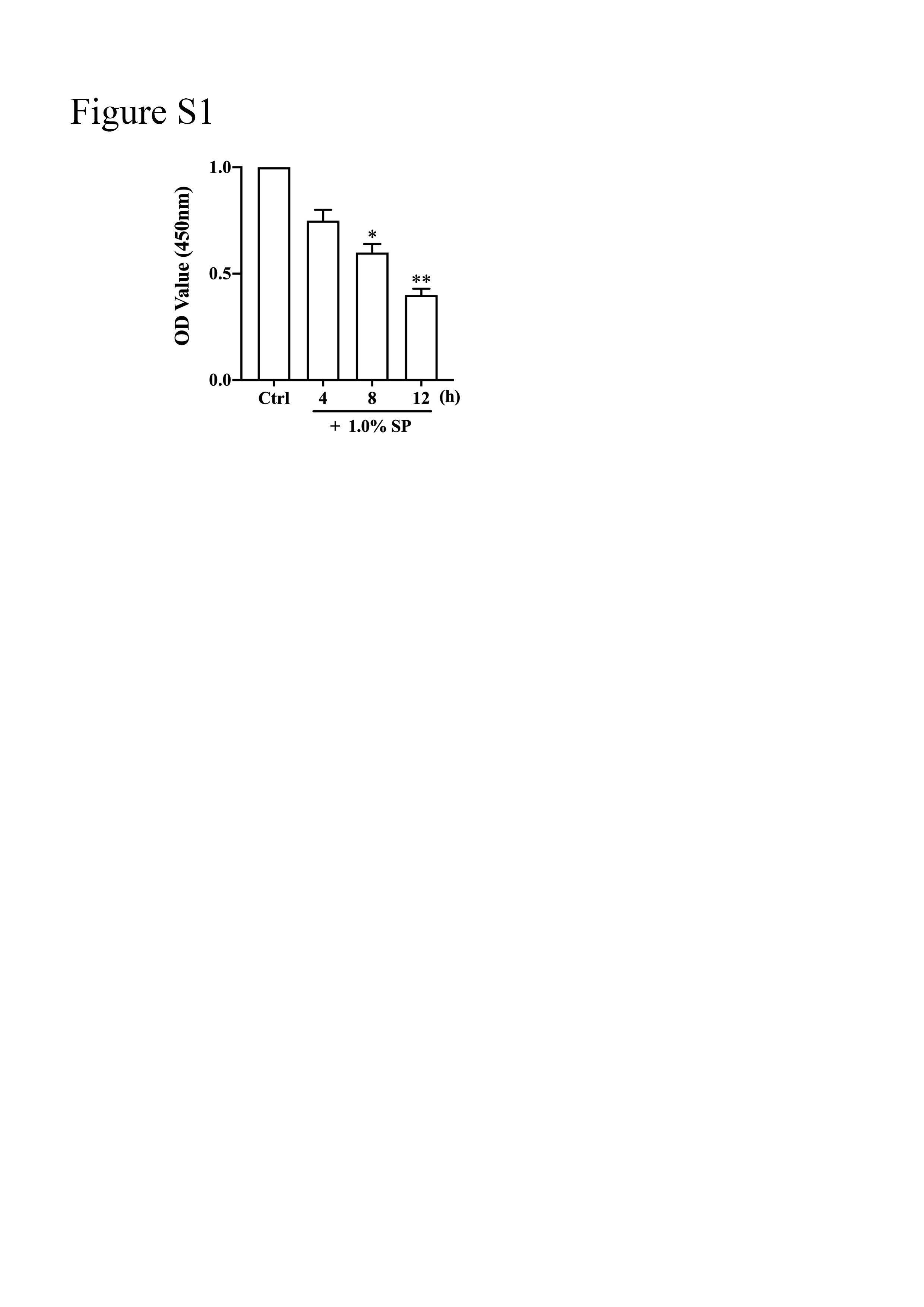

Supplement: Supplementary Figure 1 — Long-term effect of SP on cell viability. HeLa cells were incubated with 1.0% SP for the specific durations. Culture media were replaced with fresh complete media without SP. The cell viability was assessed using the CCK-8 assay 48 h after the medium replacements. Data are presented as means ± SEM of three experiments. *P < 0.05, **P < 0.01. [file Image_1.jpeg]

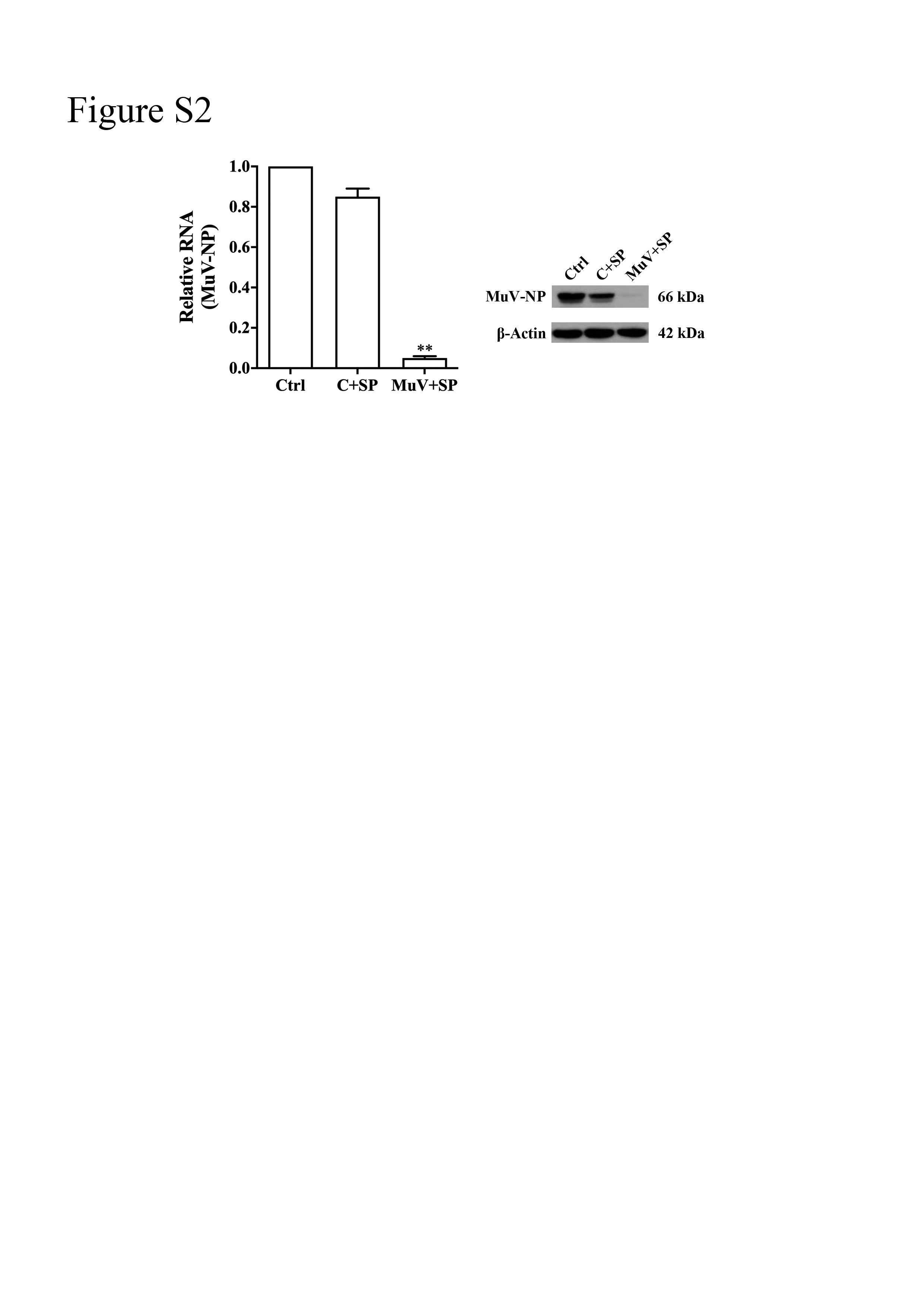

Supplement: Supplementary Figure 2 — SP effect on cells and MuV. HeLa cells were pre-incubated with 1% SP (C+SP) for 1 h and then transfected with 1.0 MOI MuV. MuV was pre-incubated with 1% SP (MuV+SP) and then transfected HeLa for 1 h. HeLa cells were transfected with MuV without SP served as the control (Ctrl). At 48 h after infection, MuV-NP RNA (left panel) and protein (right panel) levels were determined using real-time qRT-PCR and Western blot, respectively. Data are the means ± SEM of three experiments and images represent at least three independent experiments. **P < 0.01. [file Image_2.jpeg]

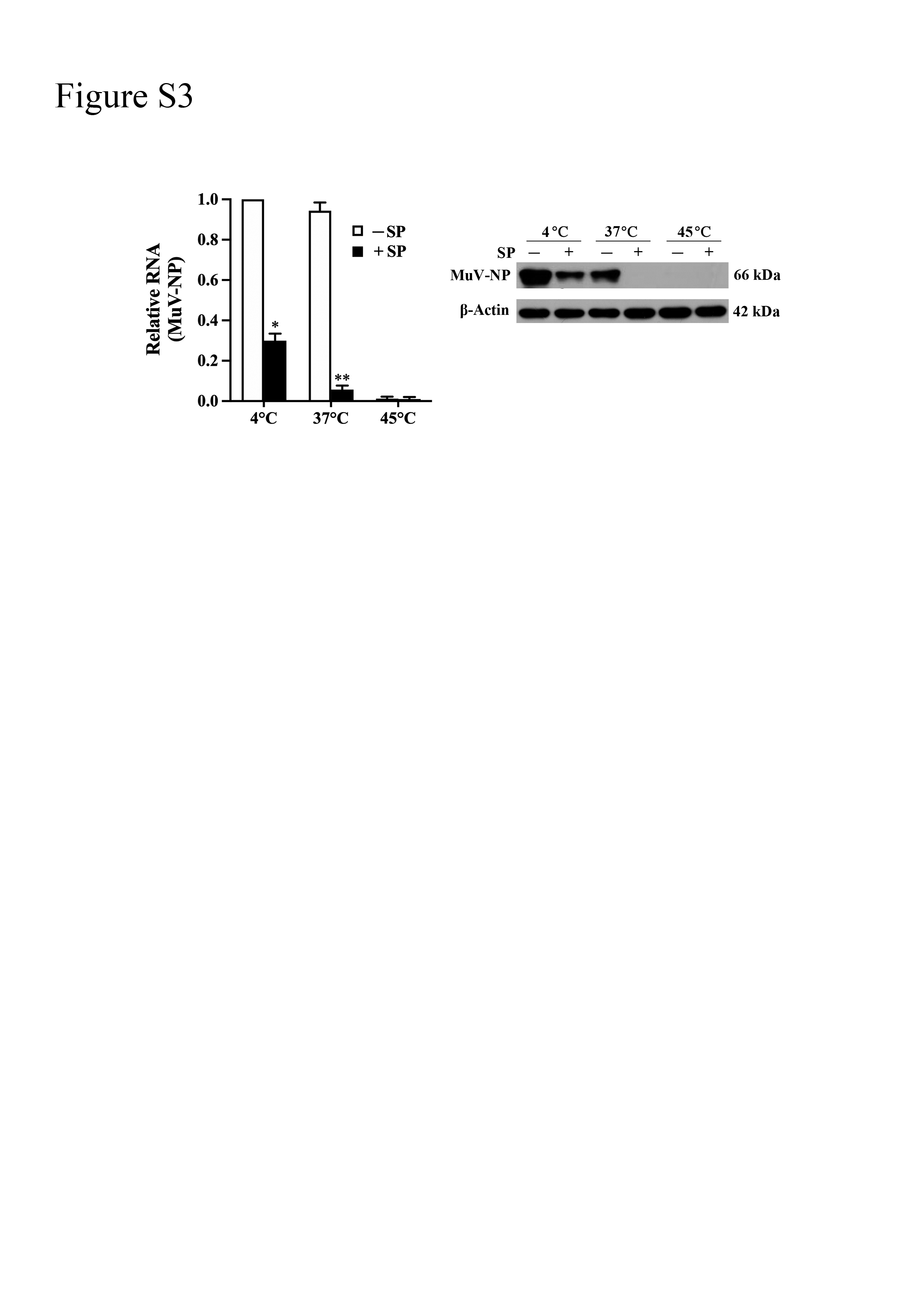

Supplement: Supplementary Figure 3 — Temperature-dependent antiviral effect of SP. MuV was incubated with 1% SP (+) or without SP (−) for 2 h at 4, 37, or 45°C. HeLa cells were infected with 1.0 MOI MuV. MuV-NP RNA and protein levels were determined using real-time qRT-PCR and Western blot. Data are the means ± SEM of three experiments. Images represent three independent experiments. *P < 0.05, **P < 0.01. [file Image_3.jpeg]

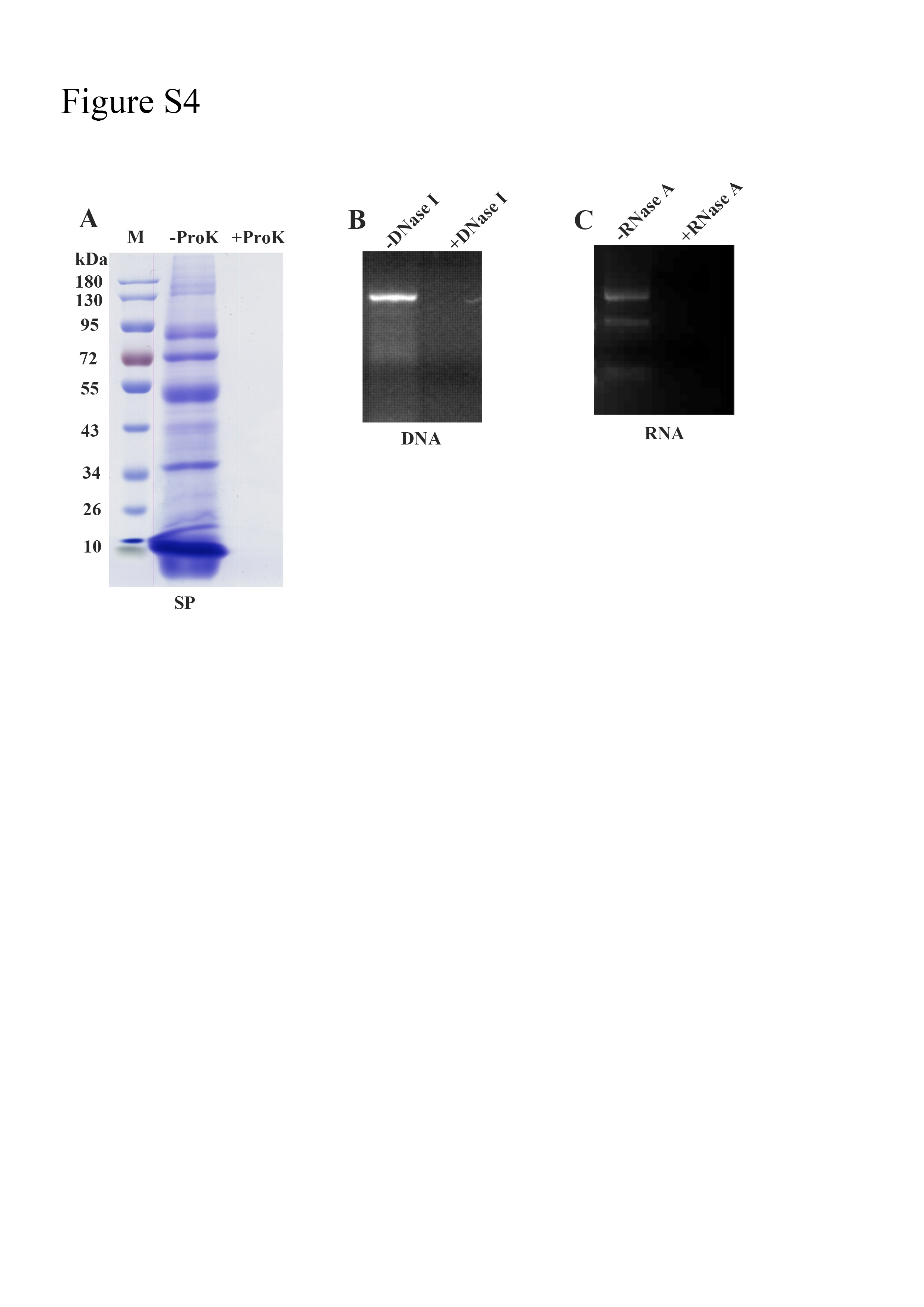

Supplement: Supplementary Figure 4 — Efficiency of enzymes. (A) Proteinase K (ProK). Seminal plasma (SP) was incubated with 300 μg/ml (+) or without (−) ProK at 37°C for 5 h and then heated in boiling water for 5 min. Each sample (2 μl/well) was loaded for SDS-PAGE with 10% polyacrylamide gel. Proteins were visualized after staining with Coomassie blue and following by decoloration. Molecular sizes were referred by protein markers (M). (B, C) Nucleic acid enzymes. Genomic DNA and total RNA were isolated from HeLa cells. DNA (B) or RNA (C) were treated with 200 U/ml DNase I or 200 μg/ml RNase A at 37°C for 5 h. DNA and RNA were determined after electrophoresis in 1% agarose. Images are the representatives of at least three independent experiments. [file Image_4.jpeg]

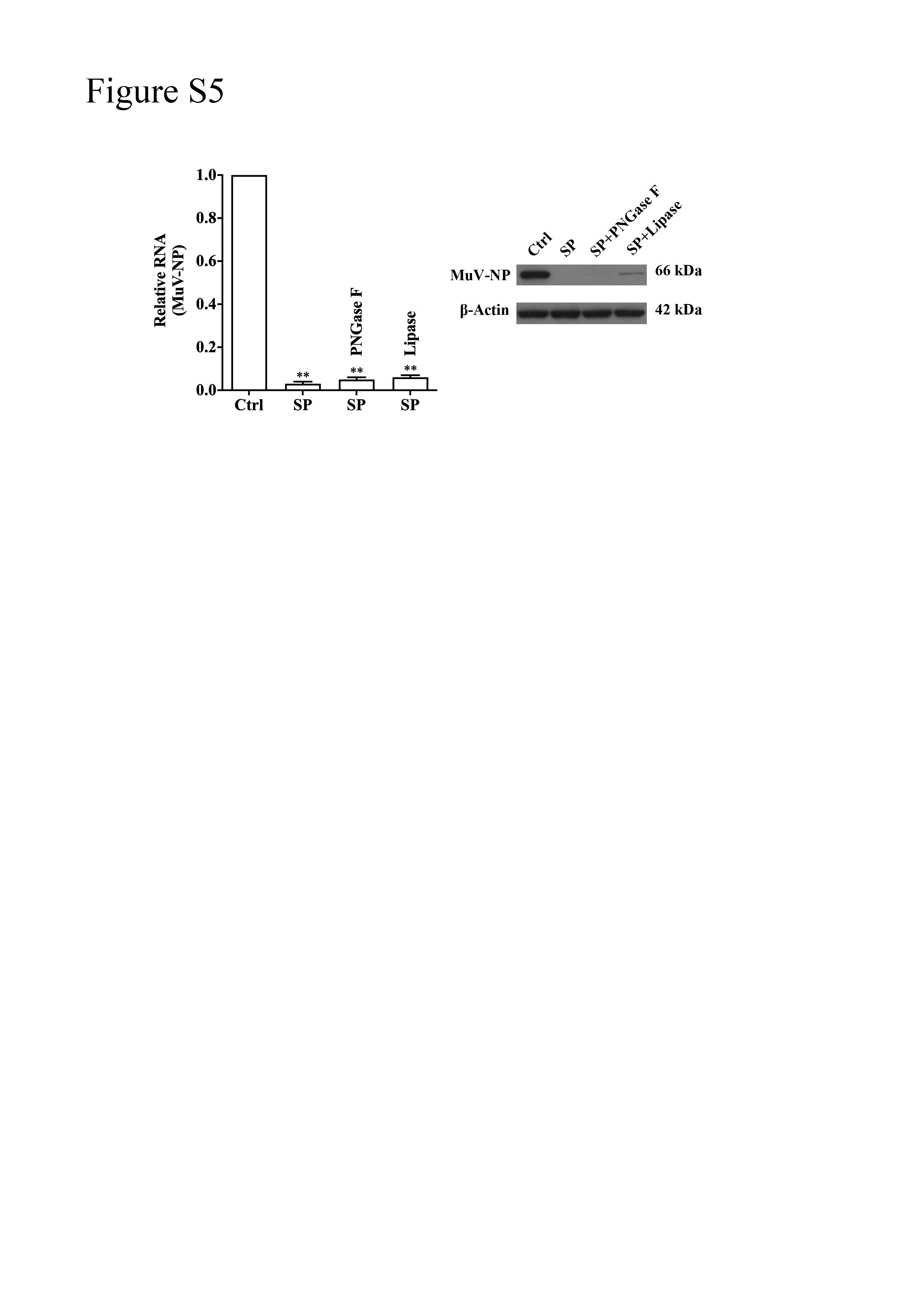

Supplement: Supplementary Figure 5 — PNGase F and lipase treatments. Seminal plasma (SP) was treated with 200 U/ml PNGase F or 100 U/ml lipase at 37°C for 5 h. MuV was incubated with SP for 2 h. MuV without SP served as the control (Ctrl). HeLa cells were infected with 1.0 MOI MuV. MuV-NP RNA (left panel) and protein (right panel) levels were determined at 48 h after infection using real-time qRT-PCR and Western blot, respectively. Images represent three experiments. Data are the means ± SEM of three experiments. **P < 0.01. [file Image_5.jpeg]

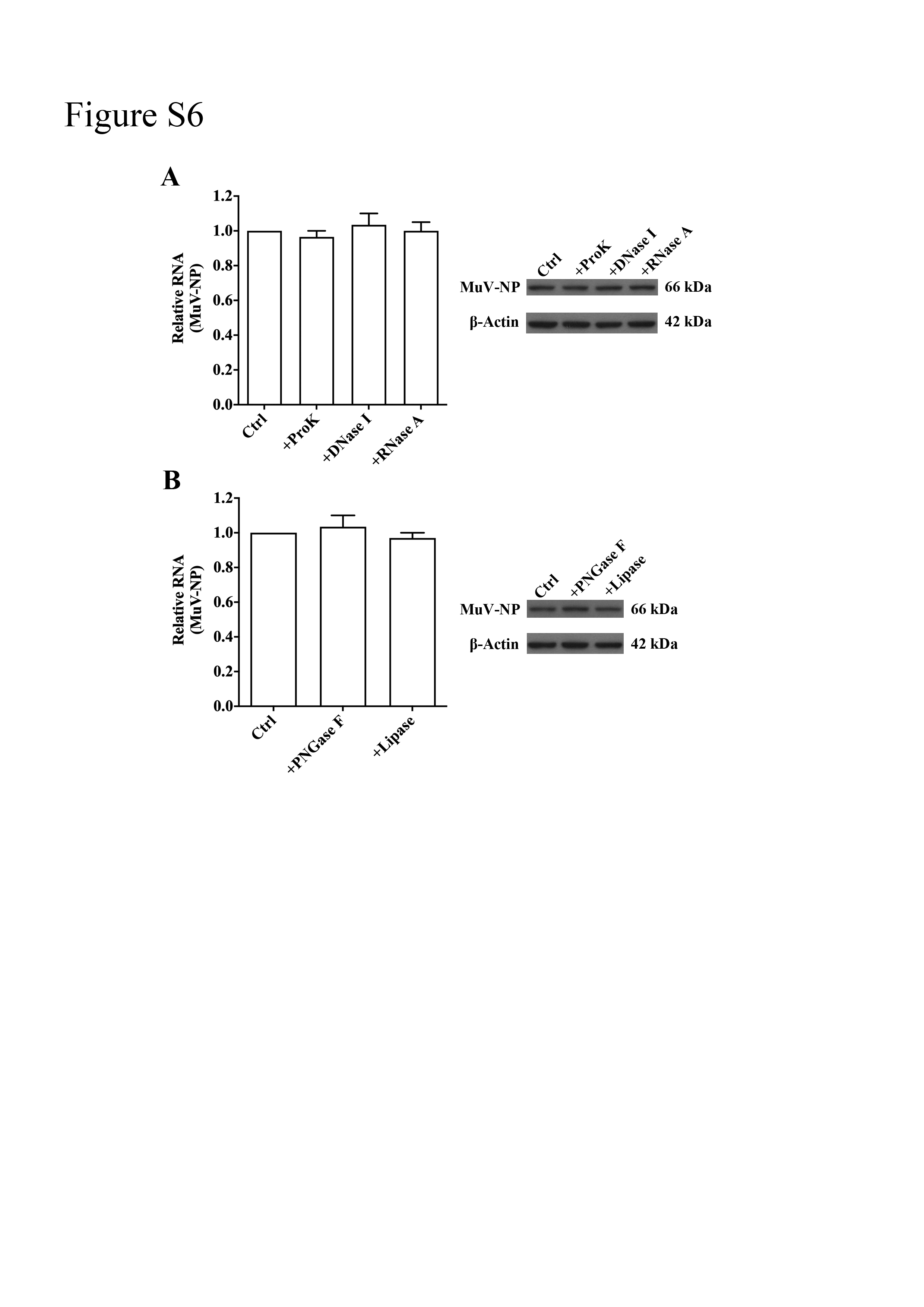

Supplement: Supplementary Figure 6 — Effect of enzymes on MuV infection. (A) Proteinase K (ProK), DNase I, and RNase A were heated in boiling water for 5 min. MuV was incubated with 300 μg/ml Pro K, 200 U/ml DNase I, and 200 μg/ml RNase A for 5 h. MuV without enzymes served as the control (Ctrl). HeLa cells were infected with 1.0 MOI MuV, and MuV-NP RNA (left panel) and protein (right panel) were determined at 48 h after MuV infection. (B) PNGase F and lipase were heated in boiling water for 5 min. MuV was incubated with 200 U/ml PNGase F or 100 U/ml lipase for 5 h. HeLa cells were infected and MuV-NP was determined as described in (A). Data are the means ± SEM of three experiments, and images are the representatives of three experiments. [file Image_6.jpeg]

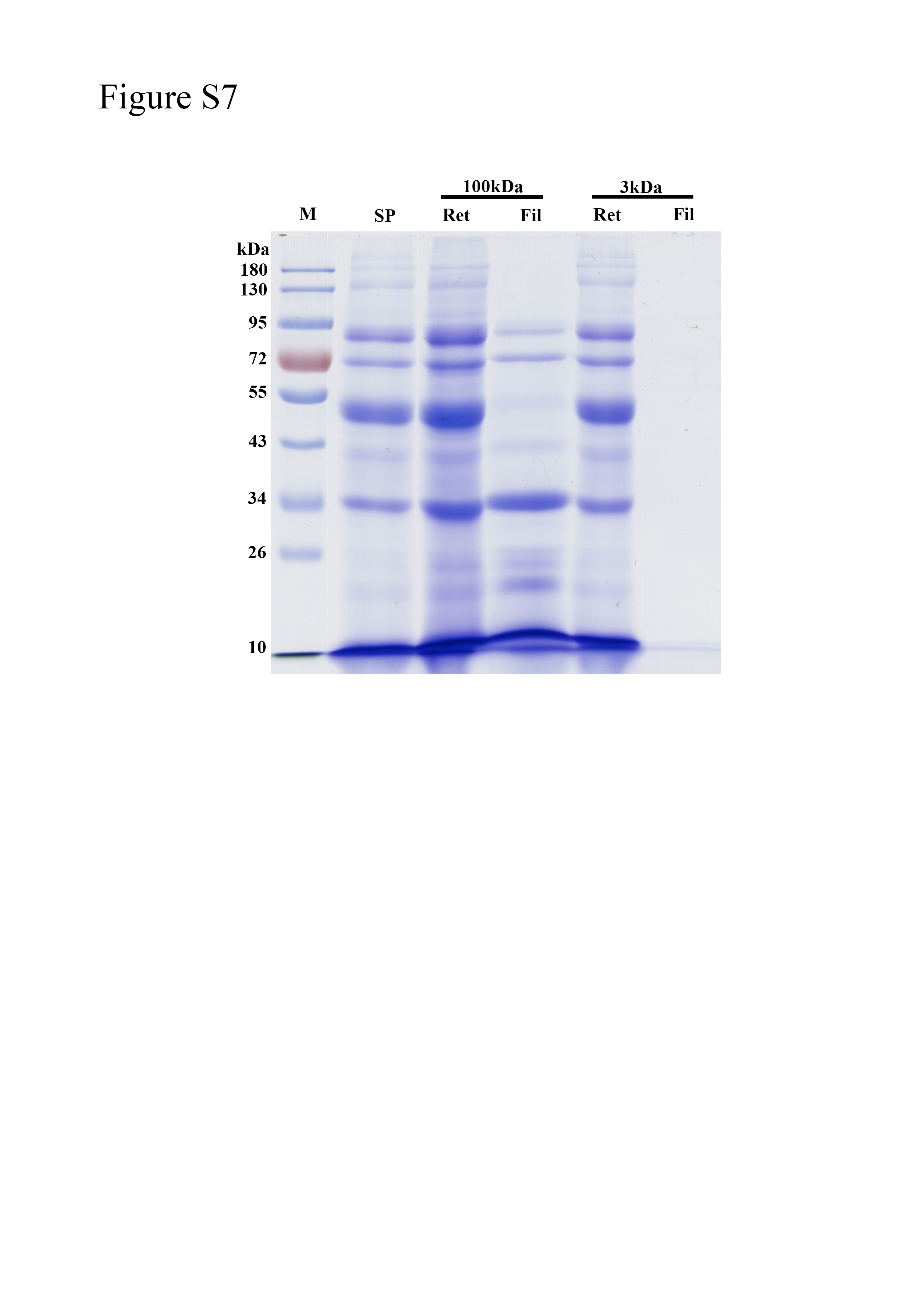

Supplement: Supplementary Figure 7 — Fractions of seminal plasma (SP). SP was separated to four fractions by ultrafiltration using 100 kDa and 3 kDa filter devices. The retentates (Ret) and filtrates (Fil) of the two filters were subjected to SDS-PAGE with 10% polyacrylamide gel. The protein components were visualized after Coomassie blue staining and decoloration. Protein sizes were referred by molecular markers (M). Images represent three experiments. [file Image_7.jpeg]
